# Exploring the Interaction Between Handedness and Body Parts Ownership by Means of the Implicit Association Test

**DOI:** 10.3389/fnhum.2021.681904

**Published:** 2021-07-07

**Authors:** Damiano Crivelli, Valeria Peviani, Gerardo Salvato, Gabriella Bottini

**Affiliations:** ^1^Department of Brain and Behavioral Sciences, University of Pavia, Pavia, Italy; ^2^NeuroMi, Milan Centre for Neuroscience, Milan, Italy; ^3^Department of Neuroscience, Max Planck Institute for Empirical Aesthetics, Frankfurt am Main, Germany; ^4^Cognitive Neuropsychology Centre, ASST Grande Ospedale Metropolitano Niguarda, Milan, Italy

**Keywords:** body ownership, IAT, handedness, asymmetry, motor behavior

## Abstract

The experience of owning a body is built upon the integration of exteroceptive, interoceptive, and proprioceptive signals. Recently, it has been suggested that motor signals could be particularly important in producing the feeling of body part ownership. One thus may hypothesize that the strength of this feeling may not be spatially uniform; rather, it could vary as a function of the degree by which different body parts are involved in motor behavior. Given that our dominant hand plays a leading role in our motor behavior, we hypothesized that it could be more strongly associated with one’s self compared to its non-dominant counterpart. To explore whether this possible asymmetry manifests as a stronger implicit association of the right hand (vs left hand) with the self, we administered the Implicit Association Test to a group of 70 healthy individuals. To control whether this asymmetric association is human-body specific, we further tested whether a similar asymmetry characterizes the association between a right (vs left) animal body part with the concept of self, in an independent sample of subjects (*N* = 70, 140 subjects total). Our results revealed a linear relationship between the magnitude of the implicit association between the right hand with the self and the subject’s handedness. In detail, the strength of this association increased as a function of hand preference. Critically, the handedness score did not predict the association of the right-animal body part with the self. These findings suggest that, in healthy individuals, the dominant and non-dominant hands are differently perceived at an implicit level as belonging to the self. We argue that such asymmetry may stem from the different roles that the two hands play in our adaptive motor behavior.

## Introduction

We all experience the solid and constant feeling of owning a body, i.e., the sense of body ownership (de Vignemont, 2011). Such experience is supposed to build upon the complex integration between interoceptive, exteroceptive, and proprioceptive signals (Tsakiris, 2010; Park and Blanke, 2019; Salvato et al., 2020a). Over the past decades, an increasing number of studies have demonstrated the role of vision, proprioception, and touch in building the sense of body ownership, through various multisensory stimulation paradigms (e.g., Rubber Hand Illusion (RHI); Botvinick and Cohen, 1998; Full Body illusion; Ehrsson, 2007; Mirror Box illusion; Medina et al., 2015; Crivelli et al., 2021). Among these, the most renowned is the RHI, which consists of administering a synchronous tactile stimulation on both the subject’s hand occluded from vision and a visible nearby rubber hand (for a review, see Riemer et al., 2019). Due to such visuotactile multisensory conflict, the subject may experience a sense of ownership toward the fake hand. This effect is typically detected via questionnaires (Longo et al., 2008) and by measuring perceptual changes, such as a shift in the perceived position of the unseen hand (proprioceptive drift). The RHI indeed demonstrates the critical role of vision and somatosensation in shaping the sense of body ownership (Botvinick and Cohen, 1998).

Another ingredient seems to play a fundamental role in building a coherent sense of body ownership: movement. For instance, Burin et al. (2015) have showed that hemiplegic patients experienced a stronger illusion when the RHI was administered on their plegic arm, suggesting that the pathological alteration of the normal flow of signals present during movements could influence body part ownership. They have also demonstrated that healthy participants experienced a stronger illusion of ownership over a fake hand after the immobilization of their arm by an orthopedic cast for 1 week (Burin et al., 2017). Crucially, the prolonged immobilization–and not the immobilization itself–produced this effect, since the strength of the illusion was similar before and after the immobilization maneuver, while it was stronger after a week of forced immobilization. These results suggest that when the involvement of a body part in motor behavior is limited for a prolonged period, either as a result of a brain lesion or forced immobilization, the feeling of ownership toward it is weakened, thus more susceptible to alterations of the sense of ownership. It is also interesting to note that in brain-damaged patients, the motor deficit (i.e., complete hemiplegia) seems to be crucial in the generation of the body ownership disorders, such as somatoparaphrenia, i.e., a delusional belief concerning the experienced disownership for the contralesional arm (Bottini et al., 2009; Vallar and Ronchi, 2009; Gandola et al., 2014a, b; Salvato et al., 2016, 2018).

Building upon this evidence, we hypothesize that humans may present a stronger sense of ownership toward body parts that play a leading role in motor behavior. There is already some evidence that different aspects of the representation of a body part may be modulated by the degree of its involvement in our motor behavior. For instance, the representation of the spatial features of the dominant hand is more stable, and thus, less susceptible to experimental manipulations, compared to its non-dominant counterpart and other body parts (Linkenauger et al., 2014). Furthermore, the visual recognition of one’s hand is faster when the target hand is the dominant (vs. non-dominant) one, and only when it is presented from an egocentric (vs. allocentric) perspective (Conson et al., 2010). These and similar variations are likely to stem from the different resolution of sensory inflow and motor outflow information associated with each body part (Linkenauger et al., 2015; Peviani et al., 2019; Sadibolova et al., 2019; Peviani and Bottini, 2020), which in turn reflects the role of that body part in our interaction with the environment (Hsiao, 2008).

Here, we put forward the hypothesis that the strength of body part ownership may vary according to the role of the body part in motor behavior, i.e., the degree by which it is involved in motor interactions. A straightforward way to test our hypothesis is measuring whether the strength of body ownership over a body part, such as the dominant hand, is predicted by the degree of its involvement in daily-life actions, which is well-captured by handedness questionnaires (Oldfield, 1971; Dragovic and Hammond, 2007; Nicholls et al., 2013).

Research addressing the role of handedness on the RHI, in which the strength of body ownership over the hand has been often inferred as inversely proportional to the susceptibility to the RHI (i.e., illusion strength) induced over the homologous fake hand, led to inconsistent findings. For instance, some works have reported that it is harder to induce alterations of the sense of ownership over the dominant hand using the RHI (Reinersmann et al., 2013; Dempsey-Jones and Kritikos, 2019). However, by using the same approach, other investigations did not replicate such pattern of findings (Mussap and Salton, 2006; Ocklenburg et al., 2011; Smit et al., 2017). It is important to remark that these works rely on the assumption that the stronger is the illusion of owning a fake body part, the weaker is the sense of ownership toward the homologous real body part (van Stralen et al., 2013).

Here, we collect a measure of ownership strength toward a body part, i.e., the degree to which the body part is implicitly represented as associated with the self, by taking a different perspective. In detail, we explored this association by means of an established and widely-used experimental paradigm, the Implicit Association Task (IAT; Greenwald et al., 1998), which has been already applied to measure the association of concepts and representations with one’s self (Bar-Anan et al., 2006; Trope and Liberman, 2010). For the first time, we use it to explore the association of a body part with the self. This approach aims to provide a more direct measure of the strength and solidity of the association between a body part and one’s self, which so far has been inferred indirectly from the temporary feeling of owning a fake body part.

We hypothesized that the strength of ownership toward a body part is modulated by the role of this body part in our motor interaction with the environment. Specifically, we expect that the strength of the association between one’s own right hand and the self (measured through the IAT) would vary as a function of the degree to which the right hand participates in daily-life actions (measured through the Handedness Questionnaire; Oldfield, 1971). Moreover, to test whether this possible association is human-body specific, and it is not explained by a broader association between the concepts “Right” and “Self,” we administered the same IAT, but addressing the association between “Right Animal Body Part” and “Self” to another group of participants.

## Materials and Methods

### Sample Size Estimation

To our knowledge, this is the first study that employed the IAT to investigate body ownership and/or handedness differences. Thus, considering the novelty of our experimental approach, we based our sample size calculation on recent work (Dempsey-Jones and Kritikos, 2019), which measured the effect of handedness on a measure of body ownership, i.e., proprioceptive drift in the RHI (Botvinick and Cohen, 1998). In this work, the authors recruited thirty-five right-handed and thirty-four left-handed, four of which were excluded from the analysis, participants (*N* = 65). We thus decided to approximate our sample size to seventy participants for each IAT’s conditions, for a total of one hundred forty participants.

### Participants

**FIGURE 1:**
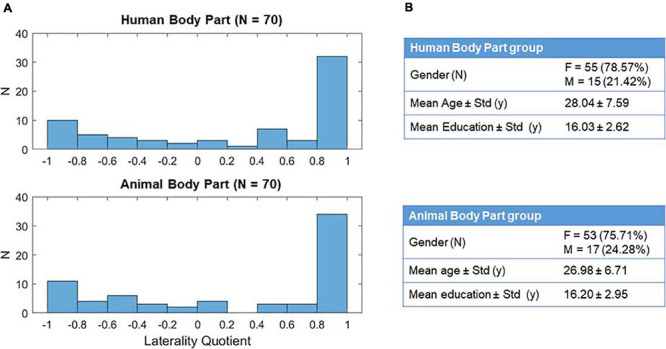
Description of the experimental sample. Panels **(A,B)** shows, respectively, the distribution of the laterality quotient and the demographic characteristics of the two experimental groups.

One-hundred-forty healthy volunteers (108 females; age range: 18–61 years old; *M* = 27.5, *SD* = ± 7.21; education: range 8–21 years; *M* = 16.1, *SD* = ± 2.80) participated in this online study. All participants were native Italian speakers, had a normal or corrected-to-normal vision, and had no previous mental or neurological illness history. The two groups of participants (Figure 1; Condition 1–Human Body Part: *N* = 70; Condition 2–Animal Body Part: *N* = 70) did not differ in age [*t*(138) = 0.866, *p* = 0.388] and education [*t*(138) = −0.361, *p* = 0.719].

Volunteers were recruited from the University of Pavia (Italy) participant database and received course credits for their participation. Right-handed and left-/mixed- handed subjects were recruited separately by explicitly targeting one population or the other in advertising the experiment. Before starting the experiment, all participants gave their informed consent by filling an online form. The experimental procedures were approved by the Ethical Committee of the Department of Brain and Behavioral Sciences of Pavia University, and they were in accordance with the Declaration of Helsinki.

### Handedness

Participant’s handedness was evaluated via online administration (using Google Modules) of the Edinburgh Handedness Inventory (EHI; Oldfield, 1971). The EHI is a self-report questionnaire composed of 10 items that assess the subject’s hand preference in performing different actions (e.g., writing, using scissors, using the fork). For each item, a strong preference for the right (R) or left (L) hand is reported by assigning 2 points to the relative hand, while the absence of a clear preference is reported by assigning 1 point to each hand. The laterality quotient (LQ) is then calculated by the formula (R - L)/(R + L). The EHI considers a subject dextral if his/her LQ is higher than 0.5; left-handed if it is lower than −0.5; mixed-handed if it is comprised between 0.5 and −0.5 included (EHI; Oldfield, 1971). In this experiment, we used the LQ as a continuous index because we were not interested in categorizing our subjects (identifying the “direction” of hand dominance), but rather, we aimed to use it as a way to measure how much more the dominant hand is used over the non-dominant one (“consistency” of handedness; Edlin et al., 2015). The participant’s laterality quotient was balanced between the two different experimental conditions [Figure 1; *t*(138) = 0.175, *p* = 0.862].

### IATs

The IAT (Greenwald et al., 1998) aims to measure the association between a target category and an attribute category. Both the IAT versions we used were adapted from Salvato et al. (2020b) and differed in terms of target and attribute categories and their respective stimuli (Table 1), which were selected to test our hypothesis. The two IAT versions were administered in a between-subjects fashion.

**TABLE 1:** The original stimuli (in Italian) and their English translations are reported for each IAT Condition and category.

	**IAT condition human body part**			**IAT condition animal body part**		
	**Categories**	**Stimuli**	**English translation**	**Categories**	**Stimuli**	**English translation**
**Target**	**Self**	*io*, *me*, and *mio*, *mia* and *miei*	*I*, *me*, and *my* (male–female–plural)	**Self**	*io*, *me*, and *mio*, *mia* and *miei*	*I*, *me*, and *my* (male–female–plural)
	**Other**	*altre* and *altri*, *loro*, *essi*, and *esse*	*other*, *others*, and *they* (male–female–synonym)	**Other**	*altre* and *altri*, *loro*, *essi*, and *esse*	*other*, *others*, and *they* (male–female–synonym)
**Attribute**	**Right human body part**	*dito destro*, *polso destro*, *nocca destra*, *dorso destro*, and *palmo destro*	*right finger*, *right*, *wrist*, *right knuckle*, *right hand dorsum*, and *right hand palm*	**Right animal body part**	*artiglio destro*, *chela destra*, *zoccolo destro*, *cuscinetto destro*, and *sperone destro*	*left claw*, *left chela*, *left plinth*, *left pad*, and *left spur*
	**Left human body part**	*dito sinistro*, *polso sinistro*, *nocca sinistra*, *dorso sinistro*, and *palmo sinistro*	*left finger*, *left*, *wrist*, *left knuckle*, *left hand dorsum*, and *left hand palm*	**Left animal body part**	*artiglio sinistro*, *chela sinistra*, *zoccolo sinistro*, *cuscinetto sinistro*, and *sperone sinistro*	*left claw*, *left chela*, *left plinth*, *left pad*, and *left spur*

One IAT version (Condition 1, Human Body Part) was designed to measure the association between the target category “Self” and the attribute category “Right Human Body Part” (Hand). In this IAT, the target categories were “Self” and “Other,” whereas its attribute categories were “Right Human Body Part” and “Left Human Body Part.” As stimuli, we selected five Italian words that are representative of each category (see Table 1 for the Italian stimuli). Regarding the target category “Self,” the stimuli were *I*, *me*, and *my*; this latter in three Italian declinations referred to a male, female or plural noun). For the target category “Other”, we selected the following stimuli: *other*, *others*, and *they*; this latter in three Italian forms (referred to a male or female noun, or synonym). Regarding the attribute category “Right Human Body Part”, the stimuli were: *right finger*, *right wrist*, *right knuckle*, *right-hand dorsum*, *and right-hand palm*. Finally, for the attribute category “Left Human Body Part,” we included: *left finger*, *left wrist*, *left knuckle*, *left-hand dorsum*, *and left-hand palm*.

The other IAT version (Condition 2, Animal Body Part) was devised to measure the association between the target category “Self” and the attribute category “Right Animal Body Part”. Its target categories (and respective stimuli) were the same as the previously described IAT (“Self” and “Other”). In contrast, the attribute categories were “Right Animal Body Part” and “Left Animal Body Part.” For each attribute category, we selected five representative stimuli. Regarding the attribute category “Right Animal Body Part,” we chose the following stimuli: *right claw*, *right chela*, *right plinth*, *right pad*, *and right spur*. As for the attribute category “Left Animal Body Part”, we included: *left claw*, *left chela*, *left plinth*, *left pad*, *and left spur*.

Each IAT (Figure 1) was composed of five blocks, each including a certain number of trials, as detailed below. In each trial, participants were required to categorize a stimulus (appearing at the center of the screen) into one out of two categories (appearing at the top-left and top-right portions of the screen). Before starting each block, participants were presented with the target and attribute categories and stimuli, informed about the locations on the screen in which the attribute and target stimuli would have been presented, and instructed to categorize them as fast as possible according to the rules of a given block (Figure 1). To categorize stimuli, participants were asked to press either the “A” or “L” key using their left or right index fingers, respectively.

In the first block (20 trials), which was the same across the two IAT conditions, participants were asked to categorize each trial stimulus as belonging to the target category “Self” (appearing on the top-left portion of the screen) or “Other” (appearing on the top-right portion of the screen). In block 2 (20 trials), participants were instructed to categorize each trial stimulus as belonging to the attribute category “Right Human Body Part” (Condition 1)/“Right Animal Body Part” (Condition 2) or “Left Human Body Part” (Condition 1)/“Left Animal Body Part” (Condition 2), appearing on the top-left and top-right portions of the screen, respectively. In block 3 (80 trials), each trial stimulus belonged to either an attribute or target category, the attribute and target categories “Self” and “Right Human Body Part”/“Right Animal Body Part” were both showed on the top-left portion of the screen, whereas the attribute and target categories “Other” and “Left Human Body Part”/“Left Animal Body Part” were both showed on the top-right portion of the screen. Block 4 (20 trials) was identical to Block 1, but the position of the target categories on the screen was reversed (i.e., “Self” was presented on the top-right portion of the screen and “Other” on the top-left portion of the screen). In block 5 (80 trials), similar to block 3, attribute and target categories were again showed together but in the opposite combination. In detail, “Right Human Body Part”/“Right Animal Body Part” were both presented associated with “Other” on the top-left portion of the screen, while “Left Human Body Part”/“Left Animal Body Part” and “Self” were both presented on the top-right portion of the screen. While Blocks 1, 2, and 4 only function as practice trials, blocks 3 and 5 are critical for the IAT, because the logic behind this paradigm is that stronger associated categories will produce faster and more accurate responses than weaker combinations. In other words, if someone presents a stronger association between “Right Human Body Part” and “Self” compared to “Right Human Body Part” and “Other” will categorize the stimuli faster and more accurately in Block 3 vs. Block 5. The order of the Blocks was fixed for all participants.

As detailed in the analysis section, from the IAT data, we computed the Greenwald et al.’s (2003), which is a measure of the strength of the association between the concept of “Self” and “Right Human Body Part” or “Right Animal Body Part” The task was programmed in OpenSesame (Mathôt et al., 2012).

### Procedure

Data collection was carried out during the Covid-19 pandemic; therefore, we recruited participants via e-mail and administered the task from remote. Each eligible participant received an e-mail containing the instructions to participate in the experiment. Before the detailed instructions, participants were required to read and fill in the informed consent. Participants were instructed to sit in front of a pc in a quiet room and minimize environmental distractors for at least 30 min, (the whole experiment generally lasted from 10 to 20 min). Afterwards, they were required to start the IAT by clicking on the link generated through JATOS (Lange et al., 2015), an open-source web platform for online studies. IAT instructions were presented at the beginning of each IAT block (see IAT section above). Finally, participants were asked to provide their demographic information (i.e., gender, age, and educational level) and fill in the EHI via Google Modules. We opted for a between-subject design to avoid a possible carry-over effect since the two IATs were, apart from the attribute’s category words, identical. Moreover, we aimed to simplify as much as possible the experimental task, considering that the experiment was administered online.

### Statistical Analysis Plan

For each participant, we computed the Greenwald’s d as follows [Greenwald et al., 2003; Salvato et al., 2020b: (Block 5 mean response times - Block 3 mean response times)/Blocks 3 and 5 pooled standard deviation]. Notably, the response times (RTs) were corrected accounting for the accuracy. RTs associated with incorrect responses were substituted with the average RT of the same block and adding a fixed penalty of 600 ms to those trials (Greenwald et al., 2003; Salvato et al., 2020b). The more positive the Greenwald’s d value is, the stronger the association between the concepts “Right human body part”/“Right animal body part” and “Self” (but also “Left human body part”/“Left animal body part” and “Other”). On the contrary, the more negative the Greenwald’s d value is, the stronger the association between the concepts “Right human body”/“Right animal body part” and “Other” (but also between “Left human body part”/“Left animal body part” and “Self”). A zero score indicates no bias at all.

To explore if handedness predicted the subject’s performance at the two IAT, we performed a linear regression for each IAT condition, with the Greenwald’s d as the dependent variable and the LQ as a continuous predictor. To directly compare whether the linear association varied between conditions, we used a general linear model to explore whether handedness modulated the IAT’s score differently across tasks. In detail, Condition (Human Body Part and Animal Body Part), LQ and their interaction were modeled as fixed effects, while the Greenwald’s d was modeled as dependent variable.

To assess that the assumptions of the linear model were not violated, we checked that the residuals of the three models were normally distributed by visually examining Q–Q plots (see Supplementary Material) and by means of the Kolmogorov-Smirnov test for normality. In all models, the residuals were normally distributed (Model Human Body Part: *D* = 0.0706, *p* = 0.852; Model Animal Body Part: *D* = 0.0490, *p* = 0.993; Combined model: *D* = 0.0401, *p* = 0.978). Concerning the last model, we checked for the assumption of variance homogeneity through Levene’s test, which was not significant [*F*(1,138) = 0.933, *p* = 0.336].

Finally, for each linear model, we reported Bayes factors (BF_10_), which represent the likelihood of the presence of the effect (H_1_) to the likelihood of the absence of such effect (H_0_), given the data. BF_10_ values larger than 1 represent evidence for the alternative hypothesis (H_1_) (Rouder et al., 2009).

Frequentist analyses were carried out using Jamovi (Sahin and Aybek, 2019; Version 1.2; The Jamovi project, 2020), while Bayesian analysis was carried out using JASP (Version 0.8.6; JASP Team, 2018).

## Results

Results showed that in the first IAT condition (Human Body Part) handedness significantly predicted the Greenwald’s d score. In detail, a significant regression equation was found [*F*(1,68) = 34.8, β = 0.582, *p* < 0.001, BF_10_ = 90611.110], with an *R*^2^ of 0.339 (see Figure 3, panel *A*).

**FIGURE 2:**
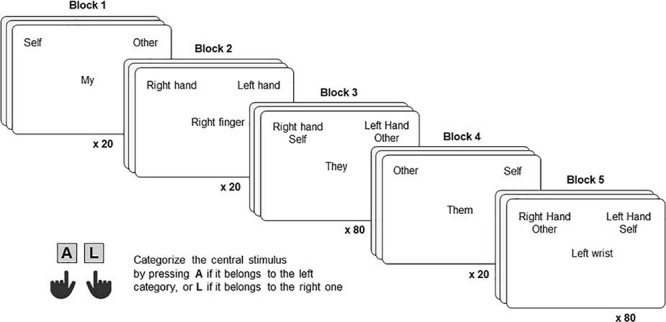
For each block of the Human Body Part condition, one exemplificative trial is represented. In each trial, participants were required to categorize the word displayed at the center as belonging to the leftward or rightward category, by pressing the “A” or “L” key, using their left or right index finger, respectively.

**FIGURE 3:**
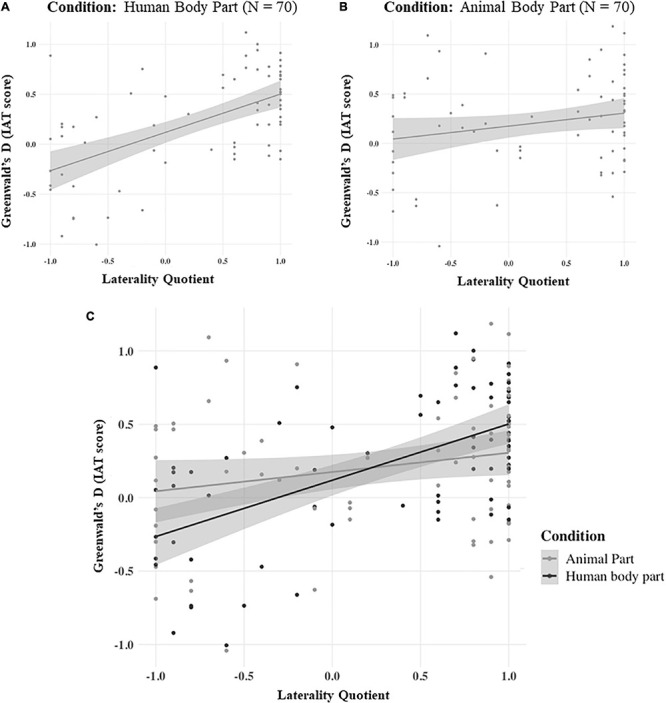
The linear relationship between the Greenwald’s d, on the Y axis, and the Laterality Quotient, on the X axis, in the Human Body Part **(A)**, Animal Body Part **(B)**, and Combined **(C)** conditions. The gray shade near each line represents the standard error of the respective model.

In contrast, in the second IAT condition (Animal Body Part) handedness did not predict the Greenwald’s d score. The linear regression model was found to be not significant [*F*(1,68) = 3.37, β = 0.217, *p* = 0.071, BF_10_ = 1.024], with an *R*^2^ of 0.0472 (see Figure 3, panel *B*).

The general linear model performed on the whole dataset [*F*(3,136) = 11.4135, *p* < 0.001, with an *R*^2^ of 0.201] revealed a significant main effect of LQ, which linearly predicted the Greenwald’s d score [*F*(3,136) = 11.4135, β = 0.4081, *p* < 0.001, BF_10_ = 14933.293]. In contrast, the main effect of Condition was not significant [*F*(3,136) = 0.0374, β = −0.0296, *p* = 0.847, BF_10_ = 0.177]. Critically, the interaction between LQ and Condition was significant [*F*(3,136) = 6.8278, β = −0.4008, *p* < 0.01, BF_10_ = 4.121], showing that the strength of handedness modulation on the IAT’s score was different when the subjects had to associate “Self” with “Right Human Body Part” or “Right Animal Body Part” (see Figure 3, panel *C*).

## Discussion

The sense of body ownership is built upon the integration between several multisensory signals and pre-acquired information about one’s own body (Tsakiris, 2010). The brain disposes of quantitatively and qualitatively varied information on different body parts, according to their role in our motor interaction with the environment. Previous research has shown that information generated by everyday movement may play a relevant role in constructing body ownership (Burin et al., 2015, 2017).

Here, we hypothesized that the degree by which a body part is represented as belonging to the self (i.e., body-part ownership) varies in the degree of its involvement in motor behavior. We expected that handedness, measured as the subjective hand preference in a series of daily-life adaptive actions (EHI; Oldfield, 1971), would predict the strength of the implicit association between the right hand and the self (measured through the IAT). In line with our hypothesis, we found that in the Human Body Part condition the laterality quotient (LQ), whose value indicates hand dominance, predicted the implicit association between “Self” and “Right Human Body Part,” measured through the IAT. More in detail, we found that the strength of this association increased as a function of the degree by which healthy subjects reported a preference for the right (over the left) hand in performing daily-life actions.

Hand dominance is associated with different aspects of human behavior, which may explain our results. For instance, it could be argued that the dominant hand is preferred over the non-dominant one simply because, semantically, the concept of “Right” is more familiar and closer to the self for dextral individuals, and vice versa for left-handers. In fact, it was shown that right-handers tend to associate “Right” with positive concepts while left-handers, in contrast, presented the opposite patterns (Casasanto, 2009). However, a purely conceptual association between “Right” and “Self” is not sufficient to explain the entirety of our data. Indeed, we found that handedness significantly predicted the association between “Right Human Body Part” and “Self,” but not between “Right Animal Body Part” and “Self” (Condition 2).

Another possible explanation for our results could be that hand dominance often reflects differences in functional brain organization. Indeed, dextral individuals present robust hemispheric lateralization for specific cognitive functions, such as language (Steinmetz et al., 1991; Badzakova-Trajkov et al., 2010) and spatial processing (Vogel et al., 2003). It could then be argued that the different IAT outcomes in right- and left-handed participants could mirror such neuro-functional asymmetries, similarly to what has been found in other body representation tasks. For instance, Linkenauger et al. (2009) showed that the dominant arm is perceived as longer than the non-dominant counterpart, only in right-handed individuals. Furthermore, right-handed individuals tend to perceive right body landmarks (e.g., the right hip) as more distant from their midsagittal plane than their left counterparts, showing poorer body exploration skills over their left (vs. right), hemibody. Crucially, such difference was not present in left-handed subjects (Hach and Schütz-Bosbach, 2010). While these works have demonstrated a lateralized pattern of performance in right-handed individuals only (Linkenauger et al., 2009; Hach and Schütz-Bosbach, 2010), we found that right- and left-handed individuals showed similar, albeit mirrored, response patterns when it comes to the sense of ownership over the dominant vs non-dominant hand. In other words, our data indicated that the association between the dominant hand with the self is similarly present in the right- and left-handed individuals (see Figure 3, panel A). Therefore, our results cannot be explained by the greater lateralization of certain neurocognitive functions in right-handed compared to left-handed individuals.

We argue that stronger ownership toward the dominant hand could be associated with its leading role in motor behavior, and that this may be adaptive to our interaction with the environment. Compared to other mammals, humans show a stronger manual preference in unimanual actions (Bryden et al., 2000; Annett, 2004). When engaged in daily-life bimanual actions, the two hands play different roles: the dominant hand has a leading role, while the non-dominant has a supporting role (Guiard, 1987; Stone et al., 2013). Many items included in handedness questionnaires (Oldfield, 1971; Dragovic and Hammond, 2007; Nicholls et al., 2013) bring examples of daily-life actions in which the two hands play very different roles (e.g., holding the scissors with the dominant hand and the paper sheet with the non-dominant one). Moreover, representational asymmetries between the two hands have been already documented. For instance, the representation of the spatial features of the dominant hand is more stable (Linkenauger et al., 2014); and such stability is possibly functional and adaptive for the dominant hand to be used as a “natural perceptual metric” (Linkenauger et al., 2013, 2014). Notably, these asymmetries pertaining to the internal representation of the hands are mirrored by asymmetries of their homologous cortical representations, involving not only the structural and functional properties of the “hand-knob” in homologous primary motor cortices, but also those of subcortical and white-matter regions (Volkmann et al., 1998; Grabowska et al., 2012; Germann et al., 2019). Indeed, the dominant hand has a crucial role in our functional interaction with the environment. A stable representation of its spatial properties and relation to the self may serve adaptive behavior.

Although quantitative observations are hampered by the low occurrence of disorders of body ownership following brain vascular accidents, our results are also coherent with the fact that these disorders seem to follow a lateralized pattern in right-handed patients. In a review considering the epidemiology of somatoparaphrenia (Vallar and Ronchi, 2009), it has been reported that the great majority of dextral patients (51/55) did not recognize their left side of the body as their own. Interestingly, the only left-handed patient showed pathological disownership toward his non-dominant hand.

Previous RHI studies exploring the role of handedness in modulating the strength of body-part ownership have produced inconsistent findings. For instance, some studies have shown that the illusion strength, measured by subjective reports and/or perceptual changes, did not vary between the dominant and non-dominant hands (Mussap and Salton, 2006; Smit et al., 2017). Other studies have reported a stronger illusion over the left hand in both right-handed and left-handed subjects (Ocklenburg et al., 2011). On the contrary, RHI susceptibility was also shown to be greater for the left hand in dextral subjects (Reinersmann et al., 2013) and to increase as a function of hand-dominance strength (Niebauer et al., 2002). Coherently, further investigations have reported a stronger RHI over the non-dominant hand in both right-handed and left-handed subjects (Dempsey-Jones and Kritikos, 2019). A possible reason for this inconsistency may be related to the intrinsic features of the RHI paradigm, which might not be well-suited to fully capture the existent asymmetry concerning the strength of ownership over the dominant and non-dominant hands. The RHI is an indirect measure of the ownership strength toward a body part, hypothesized to be inversely proportional to the susceptibility to the RHI (van Stralen et al., 2013). In other words, many RHI investigations rely on the assumption that the stronger is the illusion of owning a fake body part, the weaker is the estimated sense of ownership toward the homologous real body part. This inference can lead to informative estimates but may do not fully account for the role of the several sources of bodily information that contribute to the sense of body ownership. Some of these sources may not be necessarily affected by the RHI, such as the conscious awareness that the fake hand does not belong to the self, or the deep-rooted association of individuals’ body parts with themselves. Therefore, it is likely that the susceptibility to the RHI does not neatly reflect the strength by which a body part is associated with the self. Our work represents the first attempt to directly measure the strength of body-part ownership without inferring it from the illusory feeling of embodiment toward a fake body part. We showed that relevant aspects related to the bodily self could be unveiled not only when body ownership is artificially or pathologically altered, but also when it is healthily and fully present.

## Conclusion

By adopting an original approach to measure body ownership in healthy subjects, this study provided evidence of stronger ownership toward the dominant vs non-dominant hand. Contrarily to more traditional experimental approaches, which elicit alterations of the sense of body ownership to explore how a body part is represented as one’s own, we directly measured the degree by which a body part is implicitly associated with the self. We argue that such asymmetry may stem from the different roles that the two hands play in our adaptive motor behavior and possibly be reflected by their different cortical representations. One possible limitation of our study is that we measured participants’ handedness through a self-report questionnaire, which principal aim is to provide a trichotomous categorization of hand preference rather than obtaining fine-grained information about how much a hand is used compared to the other. Nevertheless, it is important to note that the EHI has already been used as a measure of hand dominance “consistency” in psychology and cognitive neuroscience (for a review, see Edlin et al., 2015). Future investigations could explore these aspects by using a more objective index of handedness, such as a motor performance measure (Peters and Durding, 1979; Bryden et al., 2000), while also investigating how they unfold not only in healthy but also in pathological individuals.

## Data Availability Statement

The raw data supporting the conclusions of this article will be made available by the authors, without undue reservation.

## Ethics Statement

The studies involving human participants were reviewed and approved by Ethical Committee of the University of Pavia, Department of Brain and Behavioral Sciences. The patients/participants provided their written informed consent to participate in this study.

## Author Contributions

DC and VP conceptualization, investigation, visualization, data curation, formal analysis, and writing–original draft. DC methodology. GS and GB conceptualization, project administration, methodology, supervision, and writing–review and editing. All authors contributed to the article and approved the submitted version.

## Conflict of Interest

The authors declare that the research was conducted in the absence of any commercial or financial relationships that could be construed as a potential conflict of interest.
