# Supplementary material for: Exploring the Interaction Between Handedness and Body Parts Ownership by Means of the Implicit Association Test
Source: Front Hum Neurosci. 2021 Jul 7;15:681904. doi: 10.3389/fnhum.2021.681904 (PMC8292743; doi:10.3389/fnhum.2021.681904)
Supplement: Supplementary file 1 [file Data_Sheet_1.DOCX]

Supplementary Materials for:

“Exploring the interaction between handedness and body parts ownership by means of the Implicit Association Test”

Damiano, Crivelli ^1,2*^, Valeria, Peviani, ^1,3*^, Gerardo, Salvato ^1,2,4†^, Gabriella, Bottini ^1,2,4†^

**Affiliations:**

^1^ Department of Brain and Behavioral Sciences, University of Pavia, Pavia, Italy

^2^ NeuroMi, Milan Centre for Neuroscience, Milan, Italy

^3^ Department of Neuroscience, Max Planck Institute for Empirical Aesthetics, [Grüneburgweg 14, 60322, Frankfurt am Main](https://maps.google.com/?q=Gr%C3%BCneburgweg+14+,++60322+-+Frankfurt+am+Main&entry=gmail&source=g), Germany

^4^ Cognitive Neuropsychology Centre, ASST Grande Ospedale Metropolitano Niguarda, Milano, Italy

**1. Residual plots**

In this document we provide the residuals’ distribution plot of the three linear models described in the main manuscript. In particular, it is possible to visualize the Q-Q plots of the Human body part model (Figure 1), of the Animal part model (Figure 2) and of the Combined model (Figure 3).

*
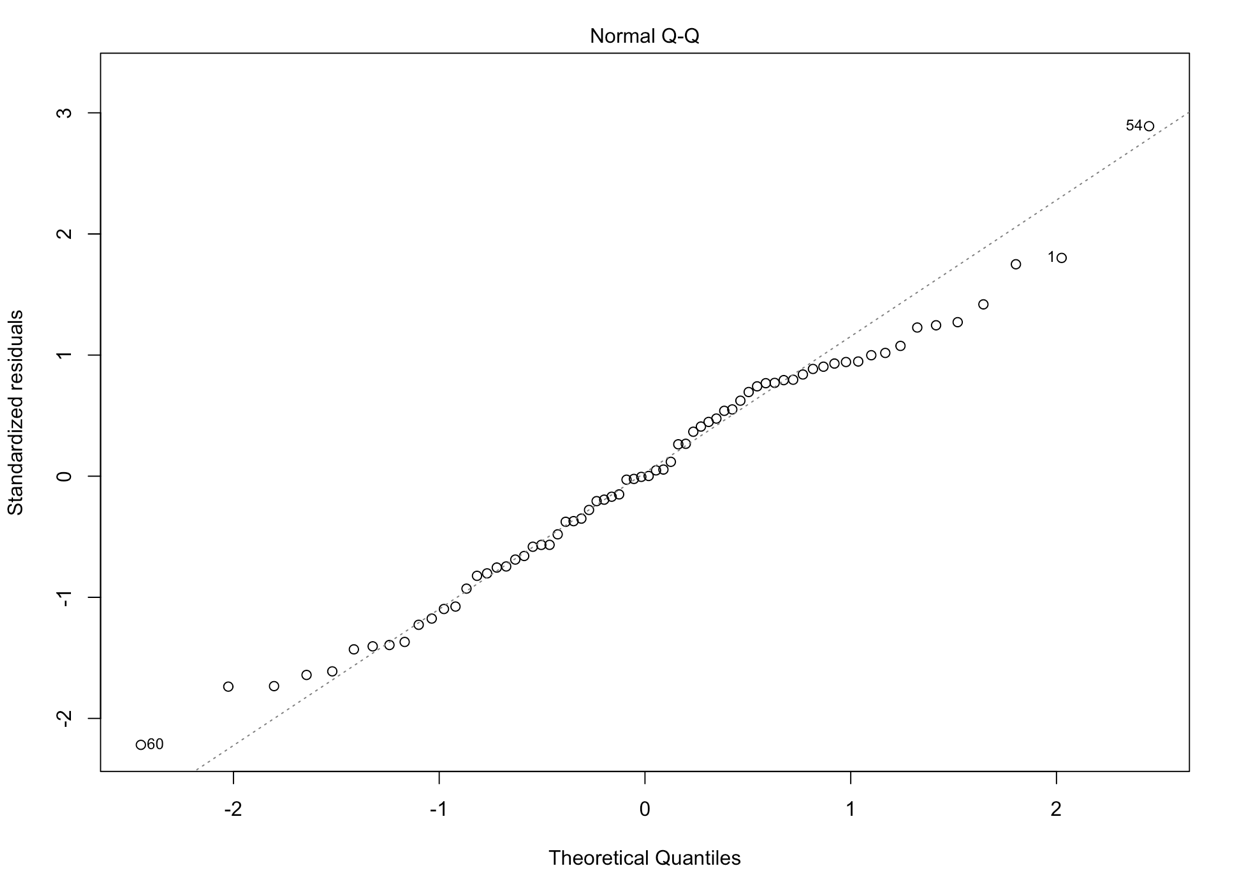
*

Figure 1. Q-Q plot of the Human body part model


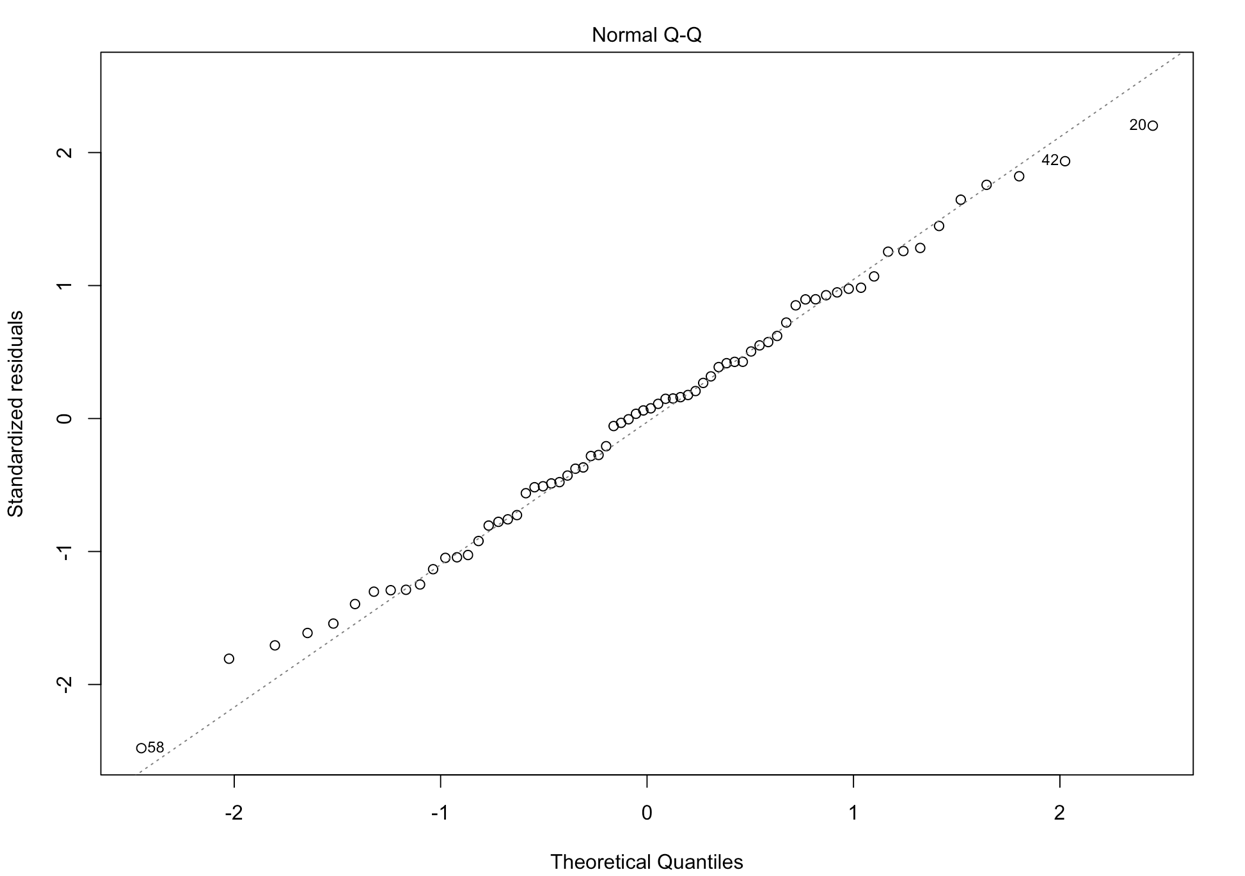


Figure 2. Q-Q plot of the Animal part model


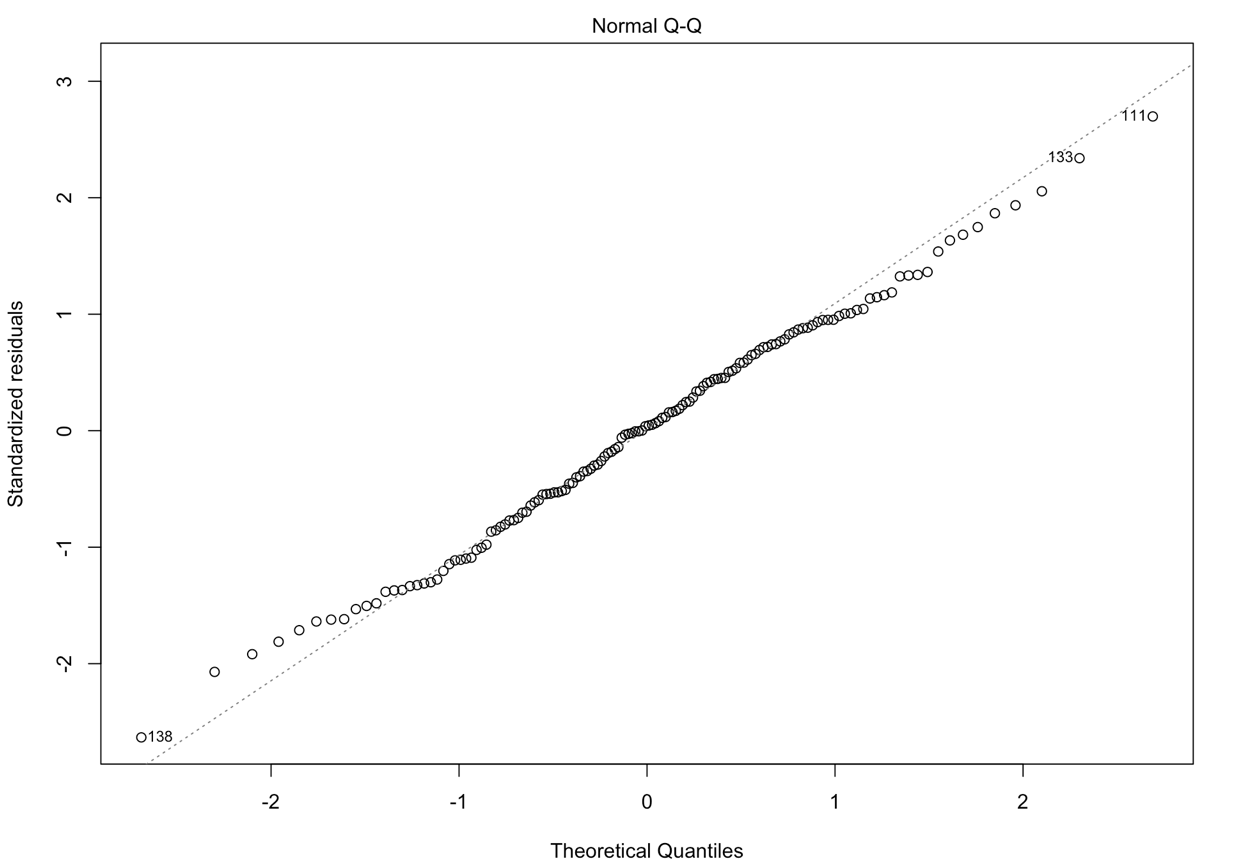


Figure 3. Q-Q plot of the Combined model

|  |
| --- |
